# Nurse Farms, Hospitals, and Private Nursing Institutions

**Published:** 1887-03-26

**Authors:** 


### Nurse Farms, Hospitals, and Private Nursing Institutions.

By a Cottage Hospital Nurse.

I have read with interest the views expressed in your paper as regards private nursing. I have been a nurse for ten years, and had experience in both hospital and private nursing. I quite agree with Mrs. Bedingfeld, that often a nurse direct from a hospital ward is quite unfit for the duties of a private nurse, "unless she is possessed of much tact and thought." To illustrate my meaning, I give an instance which came under my notice. One of our nurses was sent to nurse a wealthy lady suffering from scarlet fever. The patient had a restless night, and was feeling inclined to sleep towards morning, when the nurse, as soon as daylight dawned, drew up the blind, fetched water, and commenced to wash her unfortunate patient. The feelings of the patient can be better imagined than described. The nurse seemed quite uncomfortable, and unable to adapt herself to her new surroundings, was sent back to the hospital, and nurses from an institution were procured. I ought to explain that it was the rule of our hospital that the night-nurse put out the lights and had the washing of the patients to perform before she went off duty; and as it was a children's hospital, and a great number of patients in each ward, it was absolutely necessary to begin her work very early.

---

By the Honorary Secretary of the Workhouse Infirmary Nursing Association.

May I offer a few remarks on the discussion of private nursing institutions? Having been myself trained in a large provincial hospital which sent out nurses to private cases, and having nursed in both departments, I have seen a good deal of what I cannot help considering the drawbacks to that system. They were the following:—

(*a*) Friction between the medical staff of the hospital and the lady superintendent of the nurse's home as to the choice of nurses to be re-trained in the hospital, the temptation being great on the part of the superintendent to supply private nurses at the cost of the hospital.

(*b*) The falling short of the supply of well-trained nurses, and consequently the transfer of partially-trained probationers to the responsible charge of private patients, this frequently ending in disastrous consequences to the home, the nurse, and, most important, the patient.

(*c*) A distinctly unsettled state amongst the nurses, caused by the fluctuations of the staff from hospital to private nursing.

The London hospitals may possibly arrange so well that the drawbacks shall not be acutely felt; the second mistake certainly would not be needful; but I think that the two systems of nursing—*i.e.*, hospital and private,—totally different, and that an effort to combine the two succeeds less well than an effort to perfect each in its own particular line. Few people will refuse to admit that, as a whole, the system of hospital nursing is in a far more advanced state than that of private nursing; but there are rapid and striking improvements taking place daily in the management of the latter system, and I think it is to an advance on wholly different lines from hospitals that we must look forward.

In the first place, what Mrs. Bedingfeld has said is unanswerable—*i.e.*, that private nursing requires a certain amount of special training after the hospital course is completed; and if it were possible for all nursing institutions to supplement the usual training by three or six months' private nursing in a home for paying patients, I think a very much more finished and satisfactory private nurse would be available for the public. As it is, private patients are frequently made to feel by a nurse fresh from the hospital that they are simply "very uninteresting cases." As a lady lately remarked of her nurse sent by a hospital, "she was excellent while I was dangerously ill, but now that I am growing convalescent, she only looks out of the window all day and longs for interesting cases." I scarcely think that any doctor would consider this exactly a cheering system of nursing, but it can only be obviated by a more careful special training for private nursing, in which a nurse is taught to feel as strong an interest, but of a different kind to that she has for the "exciting cases" in a hospital. This faculty of interest in less sensational symptoms than are provided in hospitals, can only be cultivated by a refined, observant woman, and one who is not too egotistic to lose herself in her patient, even if the patient be that dread of most nurses, a "chronic case." Those private institutions which train their own nurses, seem, on the whole, to send out the best. They are chosen specially for the work, which, after all, requires a different order of character to hospital nursing,—in the latter, power of organisation, of managing numbers, great despatch in work, command over subordinates, are essential, besides other qualities, which are, of course, frequently also required in a private nurse. In one hospital which trains many private nurses, a probationer is given, towards the close of her training, the entire charge of two patients, in order that she may learn the individual care needful in private nursing; and that she may have more time to devote to these cases, she is relieved of a certain amount of ward drudgery. This, I think, is an admirable preparation for the very different sort of nursing into which she will shortly be launched; but it is an impossible system in most hospitals on account of the number of cases and the lack of workers. I may mention, in conclusion, that I consider nurses who have been for any length of time accustomed to private work only, are, as a rule, unfitted to take up hospital or infirmary work again. It is therefore desirable that nurses should carefully consider the *pros* and *cons* of the question, and not hastily decide on their future careers because of some small passing advantage. It is also desirable that the public should be "educated" as to their treatment of private nurses; but, as they are, I think every effort should be made by institutions to render the nurse thoroughly comfortable. They should feel, as far as possible, they were coming "home," when returning from cases. May I mention the Leeds Trained Nurses' Institution as setting a splendid example in this respect? I merely name it because I happen to know its management and its nurses better than any other institution of the kind in London, of which I believe the number is largely increasing.

